# Ribosomal small subunit domains radiate from a central core

**DOI:** 10.1038/srep20885

**Published:** 2016-02-15

**Authors:** Burak Gulen, Anton S. Petrov, C. Denise Okafor, Drew Vander Wood, Eric B. O’Neill, Nicholas V. Hud, Loren Dean Williams

**Affiliations:** 1 School of Chemistry and Biochemistry, Georgia institute of Technology, Atlanta, Georgia 30332, United States of America

## Abstract

The domain architecture of a large RNA can help explain and/or predict folding, function, biogenesis and evolution. We offer a formal and general definition of an RNA domain and use that definition to experimentally characterize the rRNA of the ribosomal small subunit. Here the rRNA comprising a domain is compact, with a self-contained system of molecular interactions. A given rRNA helix or stem-loop must be allocated uniquely to a single domain. Local changes such as mutations can give domain-wide effects. Helices within a domain have interdependent orientations, stabilities and interactions. With these criteria we identify a core domain (domain A) of small subunit rRNA. Domain A acts as a hub, linking the four peripheral domains and imposing orientational and positional restraints on the other domains. Experimental characterization of isolated domain A, and mutations and truncations of it, by methods including selective 2′OH acylation analyzed by primer extension and circular dichroism spectroscopy are consistent with our architectural model. The results support the utility of the concept of an RNA domain. Domain A, which exhibits structural similarity to tRNA, appears to be an essential core of the small ribosomal subunit.

The ribosome is a ribonucleoprotein complex that conducts one of life’s universal processes, which is the synthesis of proteins. The large ribosomal subunit (LSU) contains the peptidyl transferase center (PTC) and catalyzes transpeptidation. The small ribosomal subunit (SSU) contains the decoding center and reads messenger RNA (mRNA). Much of ribosomal function is performed by ribosomal RNAs (rRNAs)^1^,^2^ while the ribosomal proteins act primarily as structural stabilizers^3^. Our understanding of translation has advanced over the last decade and a half with the explosion in sequences and by the determination of three-dimensional structures^2^,^4^,^5^,^6^. X-ray crystallography and cryo-electron microscopy (Cryo-EM) have provided atomic resolution structures of ribosomes from all three primary branches of the tree of life^2^,^6^,^7^,^8^.

In ancient stepwise evolutionary processes, the ribosome acquired capabilities for RNA folding, catalysis, subunit association, correlated subunit evolution, decoding, and energy transduction^9^. Understanding function and evolution of the ribosome requires defining and recognizing secondary elements, motifs and domains. RNA domains are ruled by a special logic; folding is driven by complementary sidechains^10^, that form small, independent folds, primarily stem-loops. The RNA backbone is self-repulsive; phosphate interactions are mediated by cationic cofactors. By contrast, protein domains are large integrated units that fold cooperatively^11^, using favorable interactions between backbone atoms to create hydrophilic surfaces and hydrophobic cores^12^,^13^.

To understand and explain the ribosome and its domain structure we use the following general definition. (i) The RNA comprising a domain is compact and modular, with a self-contained and integrated system of molecular interactions. (ii) Any given RNA helix or stem-loop is contained uniquely within a single domain. (iii) Local changes such as mutations or metal binding can have domain-wide effects. (iv) Molecular interactions between stem-loops within an RNA domain dictate their orientations, stabilities and interactions to be interdependent. (v) An RNA domain has the capacity to fold autonomously when excised from the surrounding RNA.

The accepted canonical secondary structure of the SSU rRNA is based upon covariation^14^, chemical modification and RNase digestion^15^ and three dimensional structures^16^,^17^; here we are not proposing changes to the SSU rRNA historical secondary structure. We present an altered domain model for the SSU rRNA with a revised allocation of secondary structural elements to domains. We propose that an organizational hub (‘domain A’, Fig. 1) links to peripheral domains (the central domain, 3′M domain, 3′m domain, and the 5′ domain). Each peripheral domain connects to domain A by a spoke. The revised domain model differs from the historical domain model, in which the peripheral domains link directly to each other at a common origin and several helices participate in multiple domains^8^,^15^,^17^. In our revised model, with domain A as a nexus, each helix is allocated to a single domain. Here we experimentally test predictions of this domain model.

This domain model has utility, and explains some dynamical properties of the SSU. The spokes are relatively flexible, allowing the domains to move relative to each other during initiation and translocation^18^,^19^. Helix 3 is the spoke linking domain A to the 5′ domain. Helix 19 is the spoke linking the central domain while helix 28 is the spoke linking the 3′ major domain. The 3′ end of domain A is the spoke linking 3′ minor domain (Figs 1, 2, 3). Domain A incorporates the central pseudoknot (CPK)^20^,^21^,^22^,^23^ and consists of helices 1, 2, 3, 19, 27, and 28 (Fig. 3).

Domain A imposes orientational and positional restraints on the other domains, which are depicted by arcs in (Figure 2). Helices 3 and 19 of domain A form one arc and helices 27 and 28 form another arc. These two orthogonal arcs intersect within the central pseudoknot (Fig. 2b). The intersecting arcs position the four peripheral domains. Nucleotides at the 5′ end of :SSU rRNA (nucleotides 9 to 13) interact with both arcs and stabilize their relative orientation. The molecular interactions that stabilize the intersecting arcs relative to each other are illustrated in Fig. 2c,d. Universally conserved nucleotides are shown in Supplementary Fig. 6. Small changes in domain A are propagated into larger motions of the peripheral domains during translocation^19^.

One goal here is to test this domain model. Therefore we isolated domain A from the rest of the :SSU rRNA. We refer to isolated domain A as “domain A^ISO^” (Fig. 3). To form domain A^ISO^ as a single RNA polymer, we linked rRNA fragments together with three stem-loops (rGGCGUAAGCC), within helices 3, 19, and 28 (Fig. 3). The stem loops replace the connections between domain A and the four peripheral domains. The stem loops are intended to render domain A independent of the surrounding RNA without influencing its structure, especially its tertiary structure. We characterized domain A^ISO^ and mutations and truncations of domain A^ISO^ by methods including selective 2′OH acylation analyzed by primer extension (SHAPE) and circular dichroism (CD) spectroscopy. In addition, we observe that the three-dimensional structure of domain A has analogy in other biological RNAs.

## Results

### Folding of domain A^ISO^

Domain A appears to satisfy the criteria of an RNA domain. Domain A^ISO^ is characterized here by SHAPE reactivity and CD spectroscopy. We determine effects of mutations and of added Mg^2+^. We compare the SHAPE reactivity of domain A^ISO^ with that of the same rRNA elements within the intact SSU, previously published by Weeks and coworkers^24^.

Three-dimensional and secondary structures can be probed with SHAPE. Paired nucleotides, in double-stranded regions, are less reactive to the SHAPE reagent than unpaired nucleotides in loops, bulges and single strands^25^. Nucleotides involved in tertiary and Mg^2+^ interactions change reactivity upon the addition of Mg^2+ ^26,27,28,29. The data suggest that in the presence of Na^+^ alone, domain A^ISO^ forms helices 1, 2, 3, 19, 27 and 28 (Fig. 4a). For helices 1, 3 and 19, the duplex regions are unreactive and the loop regions are reactive. High reactivity of nucleotide C31 suggests a defect near the loop of helix 3. Helix 27 shows the same anomalous pattern of reactivity in domain A^ISO^ as in the intact SSU (Supplementary Fig. 8).

Helices 2 and 28 are anomalously reactive in domain A^ISO^, consistent with their anomalous reactivity in the intact SSU^24^,^30^. Nucleotides involved in base triples in the intact SSU (nucleotides G9, U20, and G22) show suppressed reactivity in domain A^ISO^. The 5′ terminus of domain A^ISO^ (which is also the terminus of the :SSU rRNA) shows elevated SHAPE reactivity as expected of unstructured RNA. Similarly, the single-stranded nucleotides between stems 3 and 19 (A45, U46, U47) have higher reactivity than the flanking stems.

Mg^2+^ ions appear to stabilize domain A^ISO^ and facilitate folding to the native state. Monovalent cations generally allow RNAs to form secondary structures and a subset of tertiary interactions. Divalent cations are required for complete folding to the native state^31^,^32^. Here we used CD spectroscopy along with SHAPE to characterize the effects of divalent cations (Fig. 5). The addition of Mg^2+^ to domain A^ISO^ increases the intensity of the diagnostic CD band at 265 nm. The intensity increases over the range of [Mg^2+^] from 0 to 700 μM after which it plateaus. These Mg^2+^ effects on domain A^ISO^ are similar to those of well-characterized globular RNAs such as tRNA^33^ and P4-P6 of the *Tetrahymena* group I ribozyme^34^.

The CD results are consistent with SHAPE reactivities. Mg^2+^ has subtle but widely distributed effects on the SHAPE reactivity of domain A^ISO^. Mg^2+^ is expected to influence SHAPE reactivities of nucleotides that directly contact Mg^2+^ or are involved in Mg^2+^-dependent tertiary interactions. This pattern of Mg^2+^-dependent SHAPE reactivity has previously been observed for tRNA, RNase P, the P4-P6 domain of the *Tetrahymena* Group I intron and Domain III of the 23S rRNA^27^,^28^,^29^,^35^,^36^. Upon the addition of Mg^2+^, nucleotides in domain A^ISO^ show slight overall decreases in SHAPE reactivity while some loop regions and bulges show increases (Fig. 4b and Supplementary Fig. 1). Reactivity of nucleotides A16 and C31 drop upon addition of Mg^2+^ suggesting that correct folding of Helix 3 requires Mg^2+^. Based on the intact SSU, A16 is expected to interact directly with a Mg^2+^ ion in the native structure^6^. Indeed, A16 shows the greatest change in SHAPE reactivity of any site in domain A^ISO^ upon addition of Mg^2+^.

### Helix 28 is an essential component of domain A

The CPK^17^ contains helices 1 and 2 (Fig. 3). We anticipated that the structure and stability of the CPK, and of domain A^ISO^, should be dependent on helix 28, because it forms a continuous stack with helix 2 in the intact SSU (Supplementary Fig. 4) and in our model of domain A^ISO^. If our model is correct, then helix 28 contributes globally to the stability of domain A^ISO^. Therefore, we have determined the effect of excision of helix 28 from domain A^ISO^.

Global changes in structure are caused by excision of helix 28. Changes in SHAPE reactivity are distributed throughout domain A^ISO^ (Fig. 4d and Supplementary Fig. 2). Reactivity increases near the 5′ terminus. Within helix 1, increases in SHAPE reactivity suggest disruption of base pairs G9-C25, A10-U24, G11-C23, U12-G22, and U13-U20 (Fig. 4d).

Furthermore, it appears that base pairing is precluded between U14 and A16 in both the intact SSU^6^,^17^ and in domain A^ISO^. These nucleotides are in a loop region in the native structure, and show a higher SHAPE reactivity than other sites in the CPK (Fig. 4a). However, when helix 28 is omitted from domain A^ISO^, U14 and A16 decrease in reactivity (Fig. 4d), suggesting non-native pairing interactions.

Domain-wide effects from the omission of helix 28 from domain A^ISO^ are revealed by CD spectroscopy. Changes in the CD spectrum of domain A^ISO^ upon addition of Mg^2+^ are diminished by excision of helix 28. Figure 5a demonstrates that changes in CD spectra after addition of Mg^2+^ are lessened by approximately 50% for domain A^ISO^ lacking helix 28 compared to intact domain A^ISO^. The diagnostic 265 nm peak does not reach full intensity in the absence of helix 28 (Fig. 5, Supplementary Figs. 5 and 9). The combined SHAPE and CD data suggest that formation of the native folded state of domain A^ISO^ is dependent on helix 28, supporting our domain model.

### A single mutation of the central pseudoknot impacts the entire domain

Pleij^22^ and Brink^23^ demonstrated that a C18A mutation within the CPK inhibits translation by affecting subunit assembly. This mutation is expected to disrupt the C18-G102 base pair. We mutated C18 to A in domain A^ISO^. This mutation is seen to cause domain-wide effects on the structure. The C18A mutation lowers the general SHAPE reactivity of the domain A^ISO^ and causes specific changes in helix 2 (U20), helix 19 (C65), helix 27 (U90) and helix 28 (G107, A108) (Fig. 4c and Supplementary Fig. 3). In addition, the unusually high SHAPE reactivities of helix 27 in domain A^ISO^ (here) are consistent with those in the assembled SSU (McGinnis and Weeks)^24^ (Supplementary Fig. 8).

The C18A mutation affects the CD spectra of domain A^ISO^. The C18A mutation, like helix 28 excision, lessens the effect on Mg^2+^ on the intensity of the 265 nm band by 50% (Fig. 5). These results indicate that domain-wide effects can be incurred by changes in sequence even if the number of nucleotides mutated is small (the C18A mutation changes 1 nucleotide while helix 28 truncation changes ~30 nucleotides compared to intact domain A^ISO^). In sum, the data appear to support our domain model of the SSU rRNA.

The structure of domain A is conserved in all ribosomes. We superimposed SSU rRNAs from bacterial and eukaryotic domains of life, including *T. thermophilus, E. coli, S. cerevisiae, D. melanogaster,* and *H. sapiens* (Fig. 6)^6^,^7^,^37^,^38^. The root-mean square deviation (RMSD) of backbone atoms of domain A in this superimposition is only 0.78 Å for ribosomes in different domains of life in the same translational state (Supplementary Table 1), consistent with a high degree of conservation of conformation. The greatest deviations are seen in the 5′ terminal region, which is single-stranded (Fig. 6). In addition, we have aligned sequences from 134 species from all three domains of life, and have calculated mutational Shannon entropies. For most of domain A, the sequences are universally conserved, with very low Shannon entropies. The sequences are most divergent in helix 3 and in the 5′ single stranded end (Supplementary Fig. 6).

## Discussion

The SSU is a central assembly of all cellular life. The architecture of the SSU has implications for ribosomal function and evolution. Here, we use high-resolution structural information to propose a domain architecture of the SSU rRNA, and have constructed an experimental system to test predictions of the domain model.

**Figure 1:**
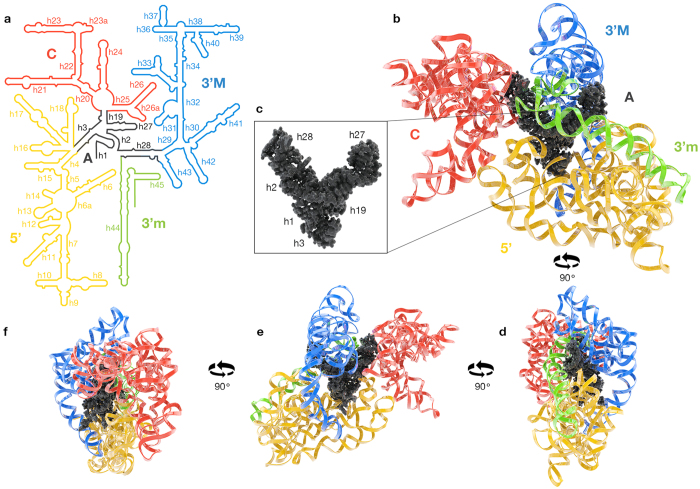
SSU rRNA domain architecture, illustrating that four peripheral domains radiate from a central core. (**a**) Secondary structure of the *T. thermophilus* SSU rRNA colored by domains. Domain A is black, the 5′ domain is yellow, the central domain is red, the 3′ major domain is blue, and the 3′ minor domain is green. (**b**) Three-dimensional structure of the SSU rRNA (PDB ID 4V51)^6^. The rRNA is represented in ribbon, except for domain A, which is in space filling representation. The domains in the three-dimensional representation are colored by same scheme as in the secondary structure. (**c**) Space filling representation of domain A alone. Helix numbers are indicated. (**d**-**f**) A series of 90° rotations of the SSU rRNA.

**Figure 3:**
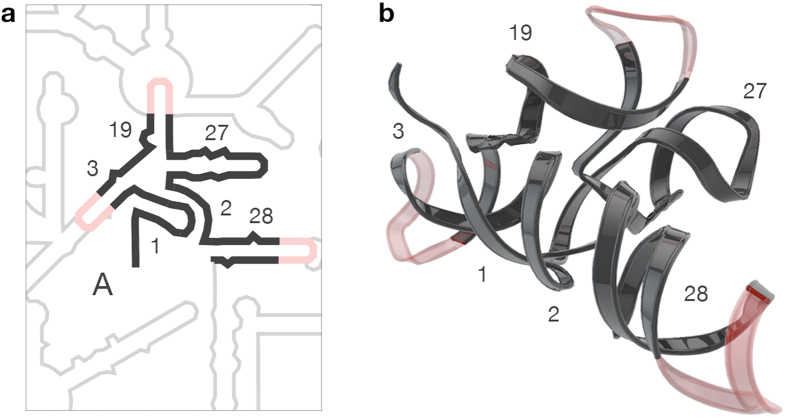
Secondary structure and three-dimensional model of domain A^ISO^. (**a**) Secondary structure of domain A. Domain A is black. The linkers that connect the domain A fragments to form a single RNA polymer are pink while the remainder of the :SSU rRNA is grey. (**b**) Three dimensional ribbon representation of domain A^ISO^ model colored as in panel (**a**). Helix numbers are indicated.

**Figure 4:**
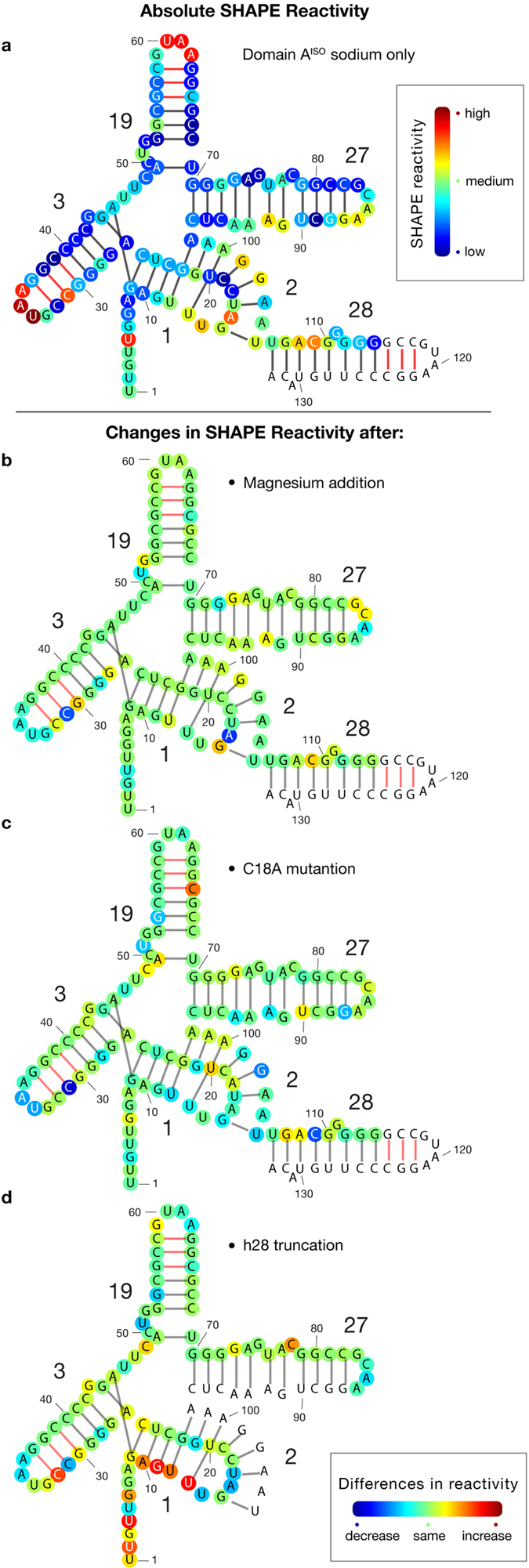
SHAPE reactivity of domain A^ISO^ mapped onto the secondary structure. Base pairs predicted from the secondary structure of the intact SSU are indicated by black lines. Base pairs in the linkers are indicated by red lines. Helix and nucleotide numbers are indicated. (**a**) Absolute SHAPE reactivity of domain A^ISO^ in the presence of Na^+^ only (250 mM). The red circles indicate high reactivity while the blue circles indicate low reactivity. The color scale is shown in the outbox. (**b**) Difference in SHAPE reactivity upon addition of Mg^2+^ (2 mM) (**c**) Difference in SHAPE reactivity upon mutation of C18 to A18. (**d**) Difference in SHAPE reactivity upon excision of helix 28. For panels b-c, red indicates a increase in reactivity, while blue indicates a decrease. Green indicates no change. The coloring scheme is shown in the outbox. Shape data for mutant and truncated domain A^ISO^ were acquired in the presence of both Na^+^ and Mg^2+^. Data were not obtained for the uncolored nucleotides. The primer binding tail is omitted for clarity. The full sequence of the construct is shown in the Supplementary Information (Supplementary Fig. 7).

**Figure 5:**
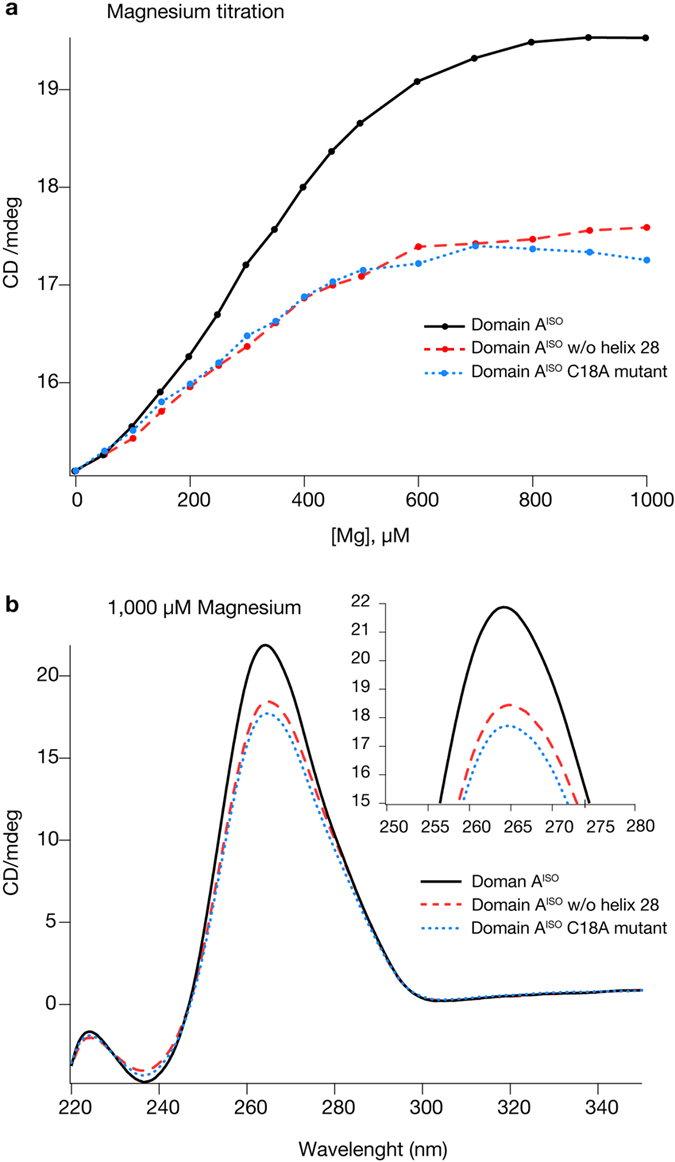
Circular dichroism spectroscopy of domain A^ISO^. (**a**) Mg^2+^ titration of domain A^ISO^ rRNA (solid black), the C18A mutant of domain A^ISO^ (dashed blue), and domain A^ISO^ rRNA with helix 28 excised (dotted red). Mg^2+^ concentration is plotted versus the intensity of the diagnostic CD peak (265 nm). (**b**) CD spectra of the same series of RNAs in the presence of 1.0 mM Mg^2+^. The outbox shows a close-up of the 265 nm peak. Initial rRNA samples were depleted in Mg^2+^ ions.

**Figure 6:**
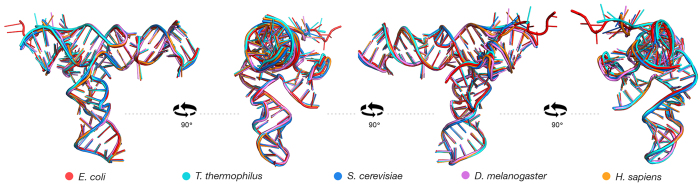
Conservation of domain A structure. Superimposition of three dimensional structures of domain A from *E. coli* (red), *T. thermophilus* (cyan), *S. cerevisiae* (blue), *D. melanogaster* (purple) and *H. sapiens* (orange).

We propose a SSU architecture in which four peripheral rRNA domains radiate from a central core, here called domain A (Fig. 1). The SSU is dendritic in structure, in contrast to the monolithic LSU. Domain A is an autonomous core at the structural and functional center of the SSU. Domain A, which includes the CPK, is a hub that connects to the peripheral SSU domains by helical spokes.

To help determine if domain A meets the formal criteria of a domain, we evaluated domain A^ISO^, an experimental model of domain A. We investigated the Mg^2+^-dependence of SHAPE reactivity and CD spectra of domain A^ISO^ and several informative sequence variants. SHAPE and CD experiments suggest compact tertiary folding of domain A^ISO^ rRNA to a near-native state in the presence of Mg^2+^ ions. A C18A mutation or excision of helix 28 causes domain-wide effects. The results of experiments described here support the integrity of domain A, and our domain architecture of the SSU rRNA. The CPK is crucial for biogenesis of the SSU, for stability of the assembled subunits, and for initiation of translation^20^,^21^,^22^,^23^.

**Figure 7:**
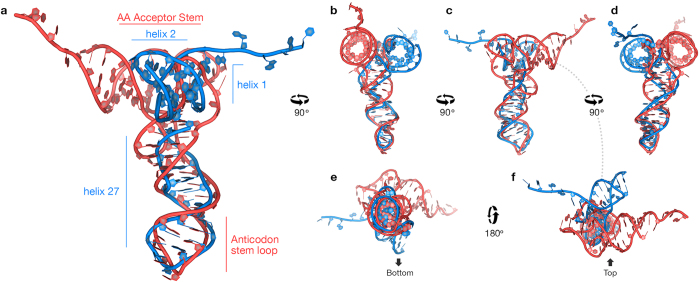
Domain A shows structural similarities to tRNA. tRNA is red and domain A is blue. (**a**) Superimposition of helices 1, 2, and 27 of domain A with tRNA (tRNA is from PDB ID: 4V51)^6^. Both domain A and tRNA form L-shaped structures. The anticodon stem-loop superimposes on the helix 27 stem-loop. View down the CCA stem of tRNA shows that it is offset from helices 1 and 2 of domain A. (**b–d**) These views show the 90° rotations on y-axis. (**e,f**) These views show 180° rotations on x-axis.

Domain A exhibits certain similarities in structure with tRNA (Fig. 7 and Supplementary Video 1). Similarities in structure to tRNA have previously been observed in select elongation factors, viral RNAs and bacterial non-coding RNA^39^,^40^,^41^,^42^,^43^. The similarity of domain A with tRNA lays in the arrangement and local conformations of helices 1, 2, and 27. Helices 1 and 2 are coaxial, and are at right angles to helix 27, giving a L-shape structure. Helix 27 of domain A is a close approximation of the anti-codon stem loop. In this region domain A is very similar to valine tRNA, with correct positioning of the CAA anticodon. However, a significant difference between tRNA and domain A is seen when helix 27 is superimposed on the anticodon stem loop; helices 1 and 2 are offset relative to the acceptor and T-stems of tRNA. Ramakrishnan previously noted a similar structural similarity in the anticodon loop of tRNA and helix 6 of SSU rRNA^44^. The 5′ end of the SSU rRNA is a rough approximation of the tRNA amino acid acceptor stem, which is formed by the 3′ end of the tRNA. The relevant nucleotides of the SSU rRNA are universally conserved (Supplementary Fig. 6) and are involved in intersubunit bridge B2c via A-minor interactions^45^,^46^. Where the CCA amino acid acceptor end of the tRNA comprises a 3′ terminus, the corresponding region of domain A core rRNA contains a 5′ terminus.

In sum, we propose a predictive model of SSU architecture by defining domain A as a hub connecting to the peripheral domains. We show that the domain concept is applicable and useful for understanding the SSU. Domain A plays a crucial role in SSU structure and function, forming a scaffold that links to each of the other SSU domains and is an evolutionary ancestor to the SSU rRNA (9). Our results support and explain previous *in vivo* and *in vitro* observations on inhibition of the protein synthesis by mutations in the CPK^22^,^23^. It has been shown that the CPK helps direct biogenesis, folding and function of the SSU. Time-resolved hydroxyl radical footprinting shows that the folding of the CPK occurs very early in subunit sythesis (10^−4^ to 10^−2^ s^−1^)^47^. Our scheme explains these results in the context of domain A, which includes the CPK. Defects in domain A impact subunit association and ultimately inhibit translation. Our results explain, on a molecular level, the effects of these mutations, which cause domain-wide changes in domain A folding as revealed by CD and SHAPE. Slight orientational alterations in helices 27-28 and 3–19 (which form intersecting orthogonal arcs) affect the overall structure, stability and dynamics of the SSU. Therefore, domain A is central player in protein synthesis machinery in all kingdoms of life.

## Methods

### Chemical reagents and synthetic oligonucleotides

The chemical reagents used here are molecular biology grade or higher. DNA primers and oligonucleotides were purchased from Operon MWG. All aqueous solutions were prepared with deionized, distilled, nuclease free water (HyClone, Thermo Scientific). For the experiments in the absence of divalent cations, nuclease free water was treated with the Chelex 100 Resin (Biorad) chelating resin and recovered with 0.2 μm Ultrafree–MC–GV Centrifugal Filters (Milipore). All the experiments are reproducible and repeated at least 2 times unless otherwise stated.

### Construction of the transcription vector for domain A^ISO^ rRNA

The *Thermus thermophilus* HB8 strain SSU rRNA sequence was obtained from NCBI database. The domain A^ISO^ gene minus helix 28 was created by recursive PCR using the four oligonucleotides (5′ to 3′):

Forward 1: GGTGTGGGAATTCTAATACGACTCACTATAGGGTTGTTGGAGAGTTTGATCCTGGCT

Reverse 2: CAGTGAATCCGGGGCCTTACGGCCCCTGAGCCAGGATCAAACTCTCCAAC

Forward 3: GTAAGGCCCCGGATTCACTGGGCGCCGTAAGGCGCCTGGGGAGTACGGCC

Reverse 4: CACCAAGCTTATTCCTTTGAGTTTCAGCCTTGCGGCCGTACTCCCCAGGC

The flanking primers were;

Forward: TGAGTCGTATTAGAATTCCCACACC

Reverse: GAAACTCAAAGGAATAAGCTTGGTG.

The domain A^ISO^ gene was cloned into the pUC19 vector using the EcoRI and HindIII restriction sites. The transformation used 5 μL of the ligation mix, which was added to 50 μL DH5α cells using the heat-shock method. Plasmids obtained by minipreps were sequenced bidirectionally by Operon MWG.

Helix 28 was added with Q5 site-directed mutagenesis (NEB) using forward AAGCTTGGCGTAATCATGG and reverse TGTACAAGGGCCTTACGG primers. The C18A mutant was also made by Q5 site-directed mutagenesis, using forward AGAGTTTGATACTGGCTCAGG and reverse CCAACAACCCTATAGTGAG primers. For SHAPE experiments, a primer binding tail was added to the 3′ end by PCR using the reverse primer CACCAAGCTTGAACCGGACCGAAGCCCGATTTGTGTACAAGGGCCTTACGGCCCCCCGTCAATTCCTTTGAGTTTCAGCCTTGC (5′ to 3′). The secondary structure of domain A^ISO^ with the SHAPE tail is shown in the Supplementary Fig. 7.

### Transcription and purification of the domain A^ISO^ rRNA

The pUC19 plasmid containing the domain A^ISO^ gene was digested with HindIII-HF (NEB) for 2 hours at 37 °C as described by manufacturer. The reaction mixture was incubated at 80 °C for 20 minutes to deactivate the enzyme. The reaction was purified with SmartSpin nucleic acid & purification columns (Denville Scientific Inc.) using DNA Clean & Concentrator Kit buffers (Zymo Research Corp.) Digested plasmid (400–1,000 ng) was used as a template for T7 RNA polymerase (NEB) transcription. Run-off transcription reaction was prepared according to manufacturer’s description (NEB T7 High Yield RNA Synthesis Kit). The reaction mixture was incubated for 16 hours at 37 °C. After incubation, 1 μL Turbo DNAse (Ambion) was added to the reaction mixture, which was then incubated for 30 minutes at 37 °C. RNA was purified by ammonium acetate precipitation. Ultimately, 40 μL nuclease-free H_2_O was added to the dried pellet and the OD was measured with a Nanodrop (Thermo Scientific). RNA was further purified by G25 size exclusion chromatography (illustra^TM^NAP^TM^-10, GE Healthcare).

### SHAPE reactions and di-deoxysequencing

Dideoxy sequencing reactions were carried out by heating a 20 μL solution of 50 ng/μL domain A^ISO^ rRNA mixed with 10 μL (0.8 μM) 5′ 6-FAM labeled GAACCGGACCGAAGCCCG primer (Operon MWG). To anneal the primer to the RNA, the reaction was heated to 85 °C and slowly cooled to 30 °C at a rate of 1.5 °C per minute. For domain A^ISO^ rRNA lacking helix 28, 5′ 6-FAM labeled TATTCCTTTGAGTTTCAGCC primer was used. After primer annealing, 20 μL mixture of SuperScript^®^ III Reverse Transcriptase (Invitrogen) reaction mixture prepared and added to domain A^ISO^ rRNA and primer mixture to give the final concentrations of 1X RT buffer, 2 mM DTT, 0.625 mM dNTPs, and 2.5 mM ddNTPs (TriLink BioTechnologies). The reverse transcription reaction was carried out by incubating 50 μL reaction mixture at 55 °C for 2 hours and quenched for 15 min by heating to 70 °C.

For the SHAPE reactions, a 70 μL solution of 150 ng/μL domain A^ISO^ rRNA was incubated for 4 min at 85 °C in the presence of 5 μM 1,2-diaminocyclohexanetetraacetic acid (Sigma) chelating agent and allowed to cool for 10 min into room temperature. This procedure depletes divalent cations from RNA. Divalent-free RNA was divided to two 32 μL samples and 4 μL of 10X folding buffer was added (500 mM HEPES pH 8.0, 2.5 M NaCl) for RNA folding with sodium. Four μL 10X folding buffer was added (500 mM HEPES pH 8.0, 2.5 M NaCl, 20 mM MgCl_2_) for RNA folding with sodium and magnesium. The sample was folded by incubated at 20 min at 37 °C and was divided two 18 μL solutions. One of the solutions was added to 2 μL of 800 mM benzoyl cyanide in anhydrous DMSO. The other solution was added 2 μL of pure DMSO for a negative background control. The reaction mixture was incubated 2 min at room temperature. The modified RNA was purified using Zymo RNA Clean and Concentrator Kit and eluted in 25 μL modified TE buffer (10 mM Tris, 0.1 mM EDTA). Primer annealing and extension reactions were as described above.

For the capillary electrophoresis, 1.5 μL of reverse transcription reaction mixture was mixed with 0.5 μL ROX-labeled DNA sizing ladder and 9 μL of HiDi Formamide (Applied Biosystems) in a 96-well plate. To denature the cDNA, the plate was incubated for 5 min at 95 °C. The mixture was resolved on a 3130 Genetic Analyzer (Applied Biosystems). Capillary electrophoresis data were processed using in-house MatLab scripts as described^28^. First, data were aligned via standard peaks and the baseline was subtracted. Sequencing peaks were matched with SHAPE data peaks. The traces were integrated and processed with a signal decay correction, and were scaled and normalized.

### Circular dichroism spectroscopy

A solution of 25 ng/mL RNA, 5 mM sodium cacodylate, pH 6.8 was titrated with either a EDTA or Mg^2+^. The RNA was titrated first with the chelator, followed by back-titration with Mg^2+^, taking CD scans on a Jasco J-810 spectropolarimeter after each addition. Four CD spectra collected and averaged, from 350 to 220 nm with an integration time of 4 seconds, bandwidth of 4 nm, a scan speed of 50 nm/min. The temperature was kept at 20 °C. RNA concentrations were kept constant for mutant and intact RNAs. Since titration of 0.5–1 mM Mg^2+^ gives a plateau for all of the RNAs in the CD scans, titration was stopped after 1 mM divalent cations added. All RNAs are present in 74 μM of nucleotides, which corresponds to strand concentrations of 0.55 μM domain A^ISO^, 0.55 μM domain A^ISO^ C18A mutant and 0.70 μM domain A^ISO^ w/o helix 28. CD experiments were repeated 3 times and results are reproducible.

### 3D Modeling and Minimization

The domain A three-dimensional structure was modeled the ribosome structure of Ramakrishnan (PDB ID:4V51)^6^. Nucleotides 1–29, 554–569, 881–929, and 1388–1396 were extracted from the crystal structure and capped by a stem-loop containing the three base pairs and a tetra loop with a sequence GCCGUAAGGC. The 3D coordinates of the stem loop were obtained from Hsiao *et al.*^29^. The stem loops were positioned as extensions of the domain A helices and connected to it by adding O3′-P bonds. The stem loops along with their two adjacent base-pairs from the domain A were subjected by the partial energy minimization, while the rest of the structure was held fixed.

### Energy Minimization

Partial minimization of the re-ligated rRNAs was performed with Sybyl-X 1.2 software (Tripos International, St. Louis, MO, USA) with the AMBER FF99 force field using an implicit solvent model with the distance dependent dielectric function D(r) = 20r. The non-bonded cut-off distance was set to 12 Å. Each system was minimized by 1,000 steps of steepest decent followed by 5,000 steps of conjugate gradient minimization.

### Superimposition

PDB IDs: 4V51, 4V9D, 4V88, 4V6W, 4V6X for *Thermus thermophilus, Escherichia coli, Saccharomyces cerevisiae, Drosophila melanogaster, Homo sapiens* were obtained from Protein Data Bank^6^,^7^,^37^,^38^.Structures were superimposed pairwise using PyMol “super” command with default settings.

### Data Mapping

SHAPE data are normalized and mapped on in-house RiboVision^48^ server using the custom data function.

### Shannon Entropy

Shannon Entropies were calculated as previously described^49^.

### Figures and Images

Figures of three-dimensional structures are prepared with PyMol or Maxon Cinema 4D with the ePMV plugin^50^. Secondary structures are obtained from in-house RiboVision server^48^. Labels are added in Adobe Illustrator or Adobe Photoshop.

## Additional Information

**How to cite this article**: Gulen, B. *et al.* Ribosomal small subunit domains radiate from a central core. *Sci. Rep.* **6**, 20885; doi: 10.1038/srep20885 (2016).

## Supplementary Material

Supplementary Information

Supplementary video

## Figures and Tables

**Figure 2:**
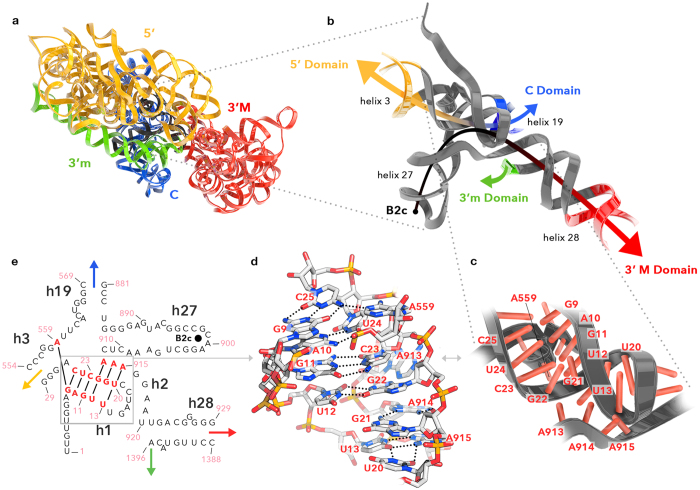
A central domain forms a scaffold for the SSU rRNA. (**a**) Three-dimensional ribbon representation structure of *T. thermophilus* :SSU rRNA colored by domains. Domain A is black, the 5′ domain is yellow, the 3′ minor domain is green, the 3′ major domain is red, and the central domain is blue. (**b**) Three dimensional structure of domain A showing the orientations of the helical spokes that radiate from it. The two perpendicular arcs indicated by the arrows, show how domain A acts a hub that organizes the SSU. (**c**) Ribbon representation showing close association between secondary elements within the domain A. Nucleotides are indicated as red sticks. (**d**) Stick representation showing some of the molecular interactions that maintain the integrity of domain A. Hydrogen bonding between the nucleotides is shown as dotted lines. Nucleotides are labeled as in panels (**c**,**e**). (**e**) Secondary structure of domain A illustrating how the other domains radiate from it. The colored arrows correspond to linkages to the peripheral domains. Helices (black) and nucleotide numbers (salmon) are indicated. The box around the red nucleotides in panel (**e**) indicates nucleotides highlighted in panels (**d**,**c**). The black dot in helix 27 of panel (**e**) corresponds the black dot at the head of the arrow in panel (**b**). This portion of helix 27 contains the bridge B2c that contacts the large ribosomal subunit. Panels (**a**–**d**) are rotated 180° relative to panels (**b**,**c**) of Fig. 1.
