# Supplementary material for: Ribosomal small subunit domains radiate from a central core
Source: Sci Rep. 2016 Feb 15;6:20885. doi: 10.1038/srep20885 (PMC4753503; doi:10.1038/srep20885)
Supplement: Supplementary Information [file srep20885-s1.pdf]

Ribosomal small subunit domains radiate from a central core

Burak Gulen[1], Anton S. Petrov[1], C. Denise Okafor[1], Drew Vander Wood[1], Eric B. O'Neill[1], Nicholas V. Hud[1], and Loren Dean Williams[1]*

[1]School of Chemistry and Biochemistry, Georgia institute of Technology, Atlanta, Georgia, United States of America 30332. *Correspondence and requests for materials should be addressed to L.D.W. (loren.williams@chemistry.gatech.edu).

Supplementary Figures

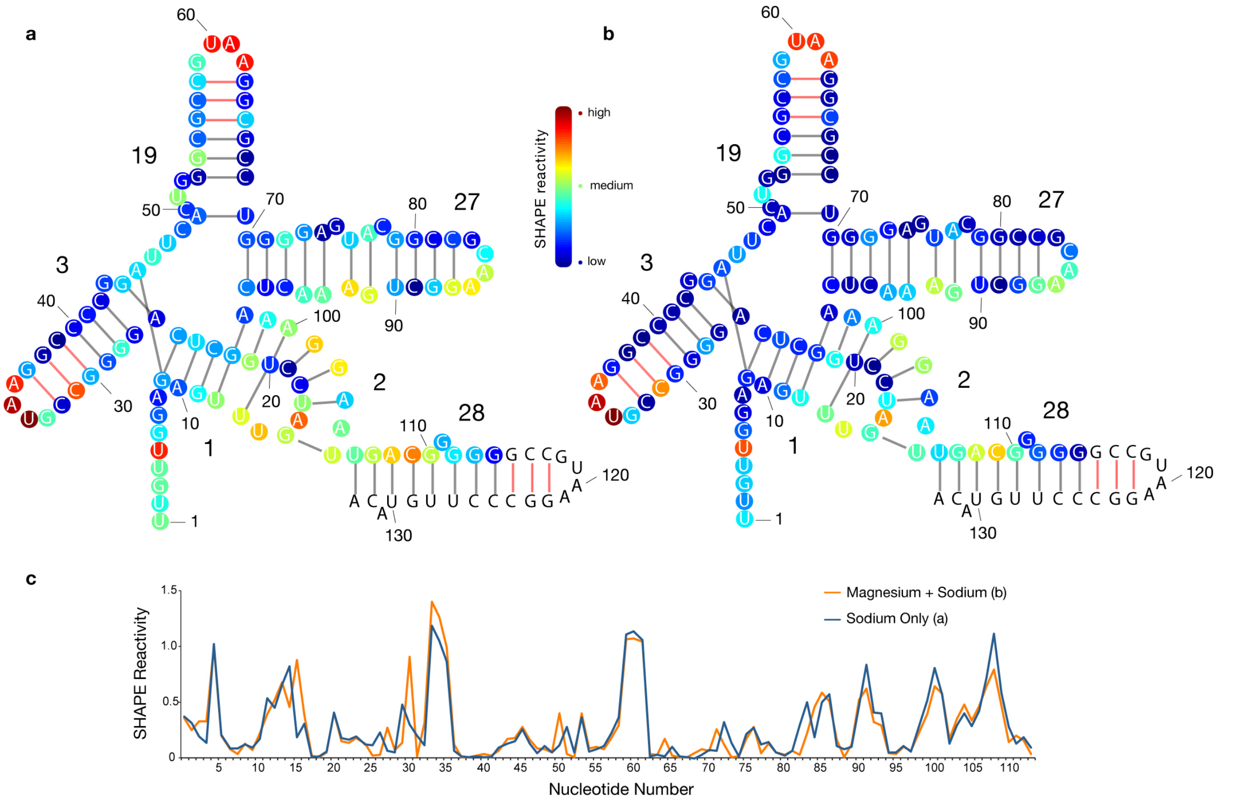

Supplementary Fig. 1 SHAPE reactivity data is mapped on the secondary structure of the Domain A^{ISO} rRNA in the absence and the presence of magnesium. The red colors indicate high reactivity while the blue colors indicate low reactivity. (**a**) SHAPE reactivity data is mapped on the secondary structure of domain A^{ISO} rRNA in the presence of sodium cations only. (**b**) SHAPE reactivity data is mapped on the secondary structure of domain A^{ISO} in the presence of sodium and magnesium cations. (**c**) SHAPE reactivity of both RNAs graphed versus nucleotide numbers. The orange data trace is domain A^{ISO} rRNA folded in the presence of sodium and magnesium whereas the blue data trace is domain A^{ISO} rRNA folded in the presence of sodium only.

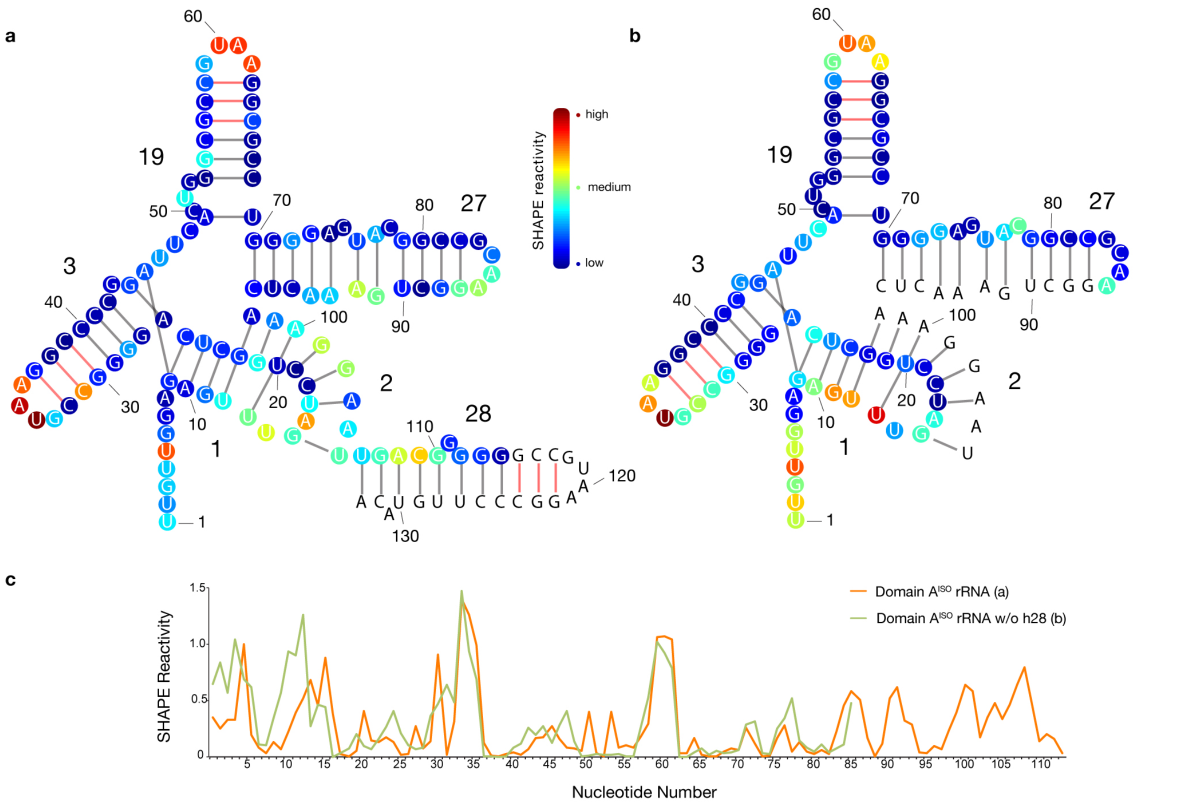

Supplementary Fig. 2 SHAPE reactivity data is mapped on the secondary structure of the Domain A^{ISO} rRNA in the absence and the presence of helix 28. The red colors indicate high reactivity while the blue colors indicate low reactivity. (**a**) SHAPE reactivity data is mapped on the secondary structure of domain A^{ISO} rRNA in the presence of sodium and magnesium cations. (**b**) SHAPE reactivity data is mapped on the secondary structure of domain A^{ISO} from which helix 28 has been excised. (**c**) SHAPE reactivity of both RNAs graphed versus nucleotide numbers. The orange data trace is the intact domain A^{ISO} rRNA whereas the green data trace is domain A^{ISO} rRNA without the helix 28.

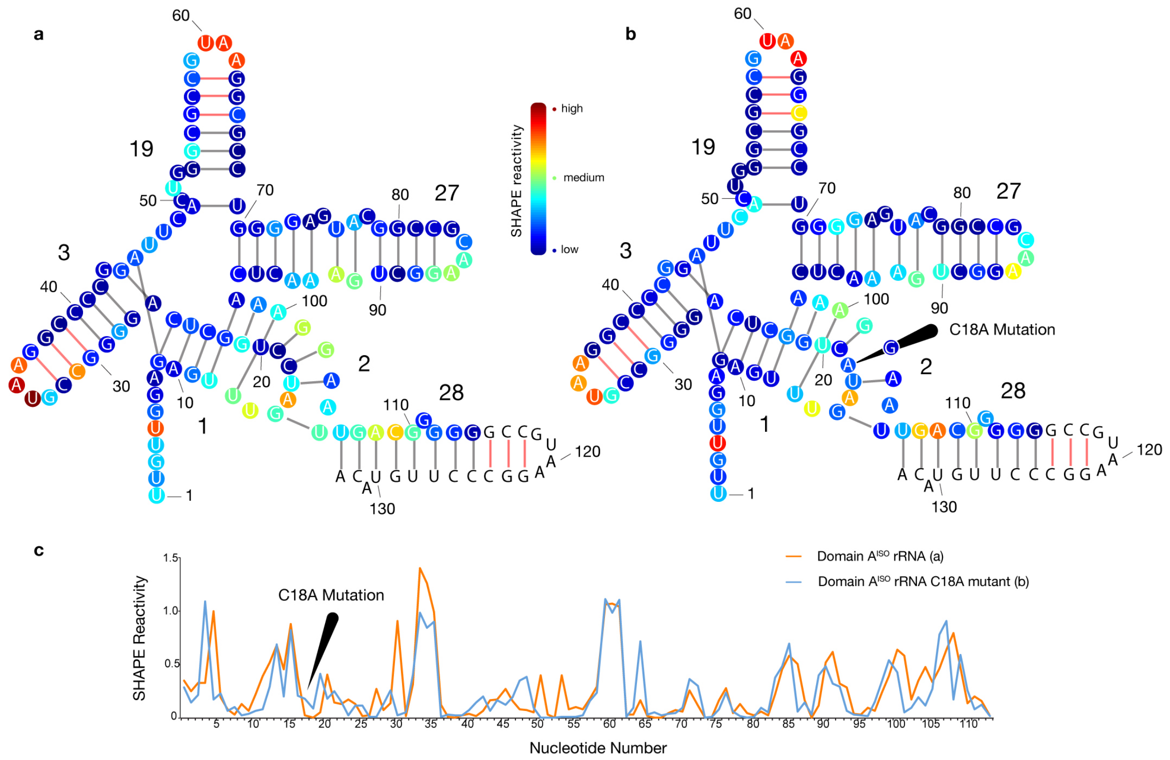

Supplementary Fig. 3 SHAPE reactivity data is mapped on the secondary structure of the Domain A^{ISO} rRNA in the absence and the presence of C18A mutation. The red colors indicate high reactivity while the blue colors indicate low reactivity. (**a**) SHAPE reactivity data is mapped on the secondary structure of the intact domain A^{ISO} rRNA. (**b**) SHAPE reactivity data is mapped on the secondary structure of C18A mutant domain A^{ISO} rRNA. (**c**) SHAPE reactivity of both RNAs graphed versus nucleotide numbers. The orange data trace is the intact domain A^{ISO} rRNA whereas the green data trace is domain A^{ISO} C18A mutant rRNA. Mutation is pointed out with black arrow.

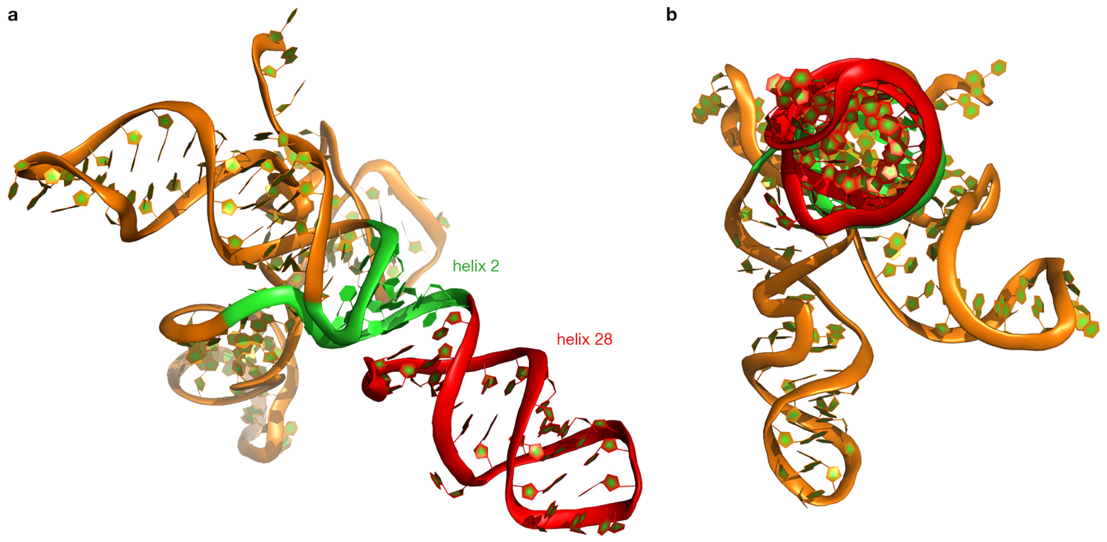

Supplementary Fig. 4 Stacking interactions between helix 2 and helix 28 of domain A^{ISO} (and 16S rRNA). (**a**) Three dimensional model and cartoon representation of domain A^{ISO} rRNA indicating continuous stack between helix 2 and helix 28. Helix 2 is shown green and helix 28 is shown red while the rest of the molecule is orange. (**b**) Cartoon representation of the same rotated model to demonstrate continuous stack through the helical axis.

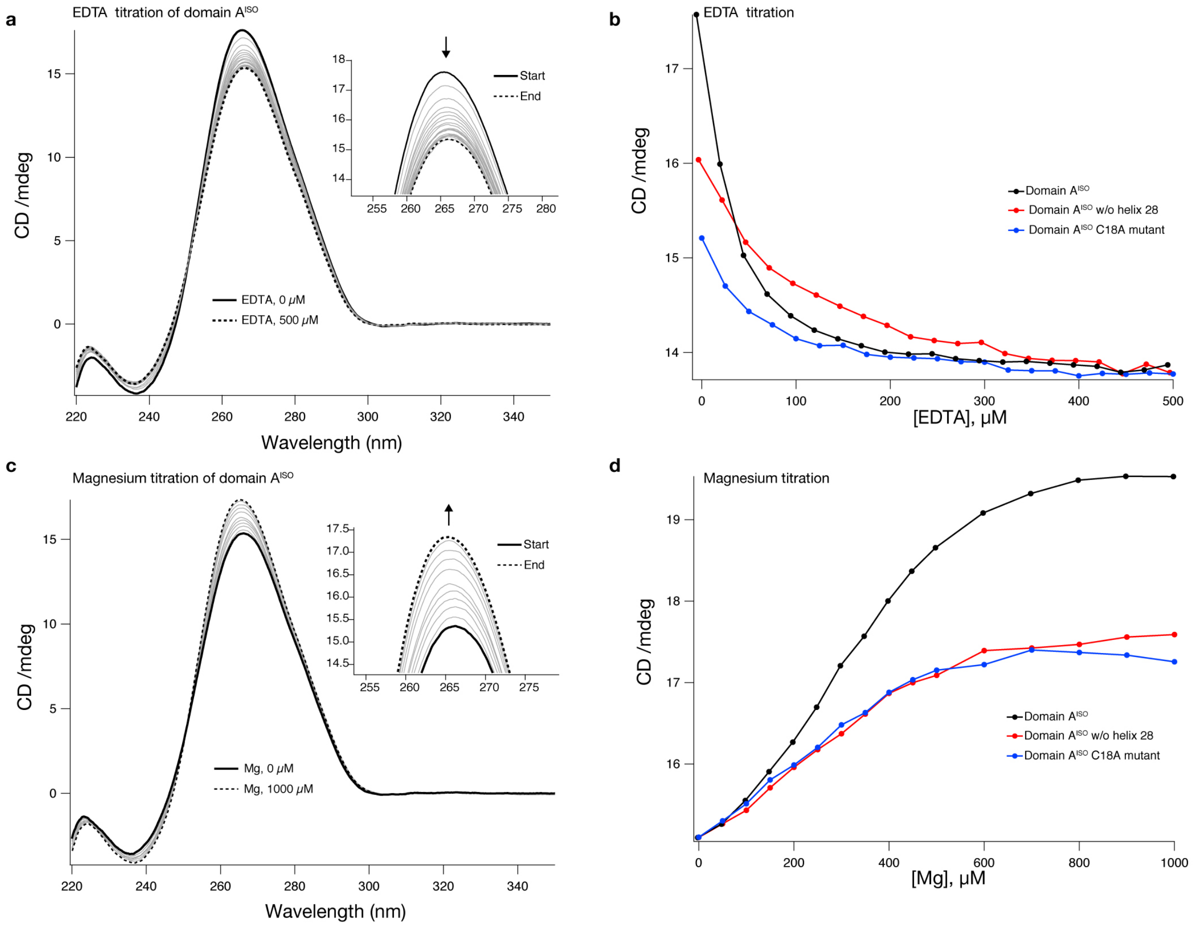

Supplementary Fig. 5 EDTA and Mg^{2+} titrations of domain A^{ISO} rRNA. (**a**) To ensure that all Mg^{2+} is removed we preformed a titration with EDTA. EDTA titration of the intact domain A^{ISO} rRNA monitored by CD. The outbox is close-up of the change at 265nm. (**b**) Graph demonstrates EDTA titration of the intact domain A^{ISO} rRNA (black), helix 28 truncated domain A^{ISO} rRNA (red), and domain A^{ISO} rRNA C18A mutant (blue). EDTA concentration is plotted versus the intensity of the diagnostic CD peak at 265 nm. (**c**) Mg^{2+} titration of divalent depleted intact domain A^{ISO} rRNA along with CD scans in each data point. Outbox is close-up of 265nm band. (**d**) Graph demonstrates Mg^{2+} titration of divalent depleted intact domain A^{ISO} rRNA (black), helix 28 truncated domain A^{ISO} rRNA (red), and domain A^{ISO} rRNA C18A mutant (blue). Mg^{2+} concentration is plotted versus the intensity of the diagnostic CD peak at 265 nm.

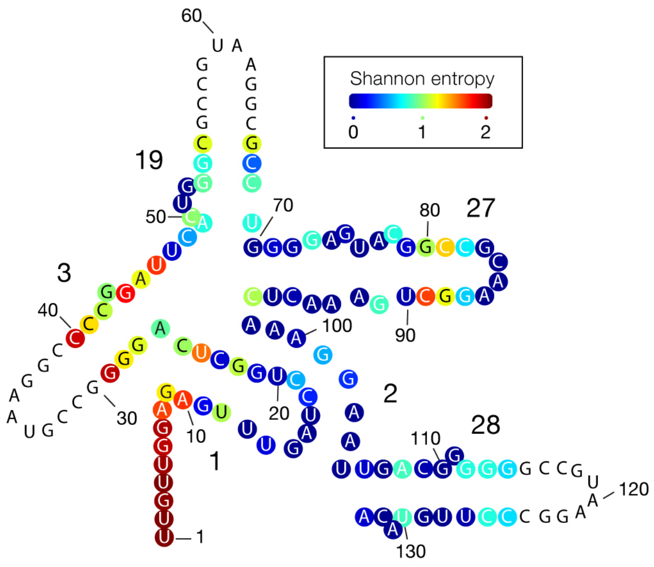

Supplementary Fig. 6 Shannon entropy is mapped on domain A rRNA secondary structure map. Dark blue circles indicate universally conserved nucleotides while red circles indicate variability. Nucleotide and helix numbers are shown in the figure.

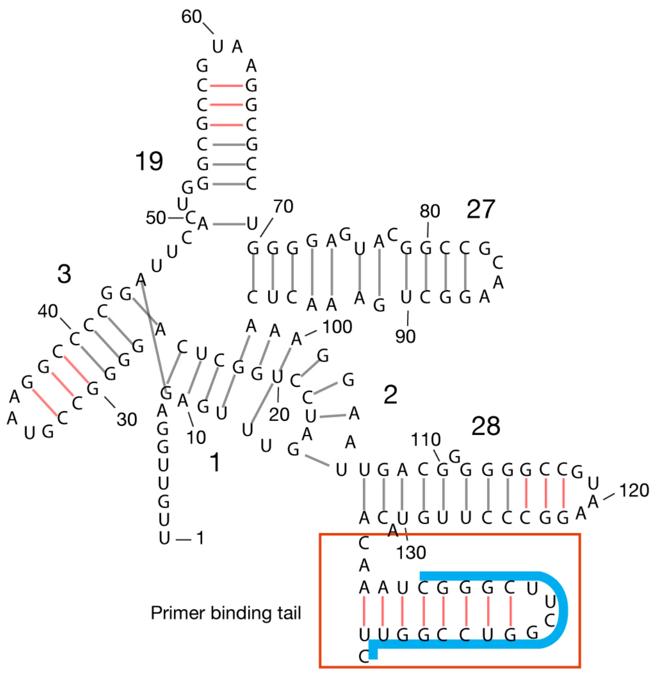

Supplementary Fig. 7 Secondary structure map of domain A^{ISO} rRNA SHAPE construct. Helix and nucleotide numbers are indicated. Base pairs are shown as black lines while predicted base pairs for our experimental stem loops and primer-binding tail is shown as red lines. Primer-binding tail helix is demonstrated in red rectangle. 5' FAM fluorescence labeled primer annealing region is indicated as blue tick line.

Supplementary Table 1 | Pairwise superimposition of domain A rRNA

RMSD	TT	EC	SC	DM	HS
TT					
EC	0.566				
SC	0.645	0.831			
DM	0.981	0.887	0.905		
HS	0.644	0.821	0.689	0.830	

Average RMSD = 0.827

Average RMSD = 0.778 (P atoms only)

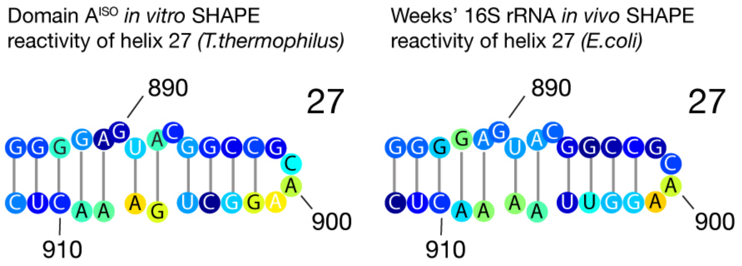

Supplementary Fig. 8 Comparison of SHAPE reactivity between domain A^{ISO} helix 27 and Weeks' intact 16S rRNA helix 27. Domain A^{ISO} helix 27 *in vitro* SHAPE reactivity from *T.thermophilus* is consistent with Weeks' *in vivo* 16S rRNA from *E.coli*. Nucleotide numbers follow the *E.coli* numbering scheme. Base pairs are indicated by black lines.

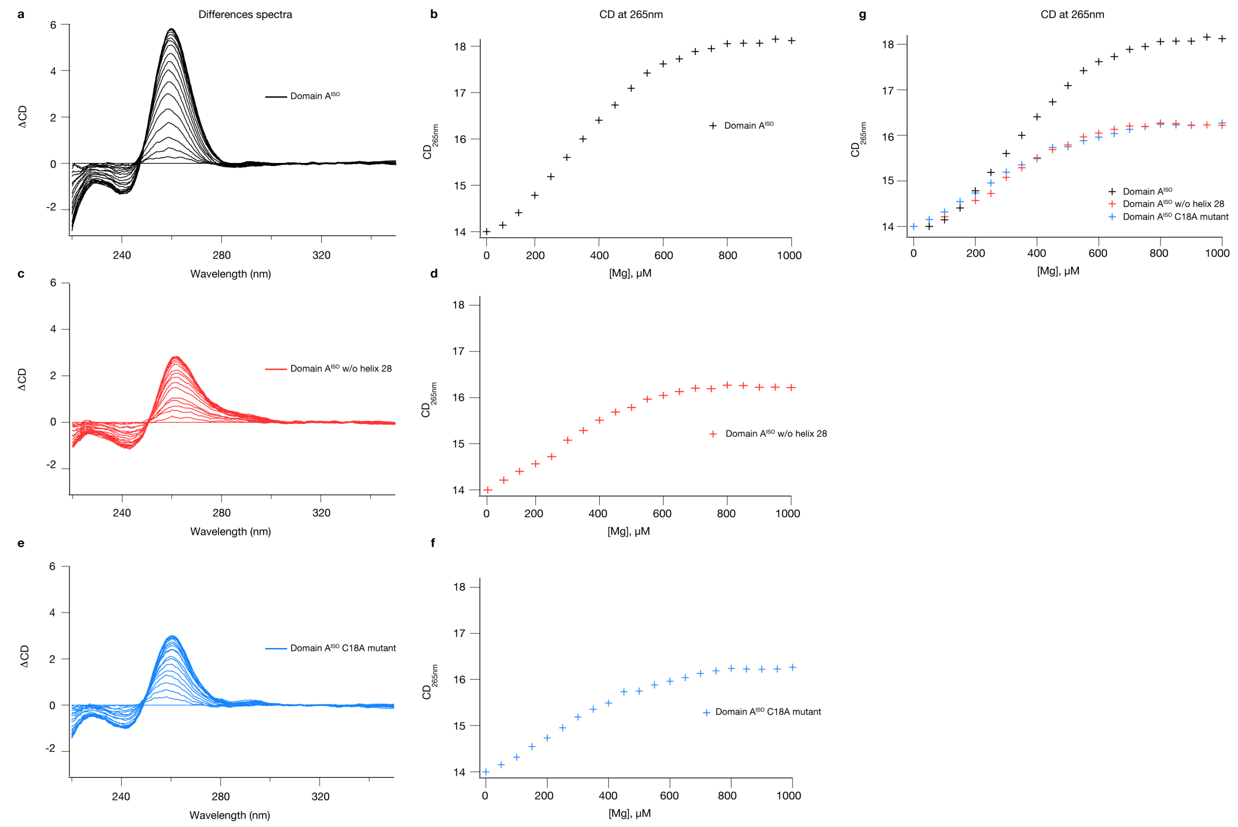

Supplementary Fig. 9 Titration up to 1 mM $MgCl_2$ of Domain A RNA and variants was monitored using CD. 500 µM EDTA was first added to all RNA samples. The magnitude of the peak at 265 nm, monitored as function of added $MgCl_2$, is shown for (**a**) Domain A^{ISO}, (**c**) Domain A^{ISO} w/o helix 28 and (**e**) Domain A^{ISO} C18A mutant. Difference spectra, obtained by subtracting the CD spectrum of the starting sample (i.e RNA sample with with 500 µM EDTA and no Mg^{2+} added) from all subsequent CD spectra obtained after $MgCl_2$ addition were obtained for (**b**) Domain A^{ISO}, (**d**) Domain A^{ISO} w/o helix 28 and (**f**), Domain A^{ISO} C18A mutant. (**g**) Comparison of peak magnitudes at 265 nm and difference spectra of Domain A RNA variants reveal that Mg^{2+} addition induces a greater change in Domain A compared to the other RNAs. All RNAs are present in 74 µM of nucleotides, which corresponds to strand concentrations of 0.55 µM Domain A^{ISO}, 0.55 µM Domain A^{ISO} C18A mutant and 0.70 µM Domain A^{ISO} w/o helix 28.

Supplementary Video 1 Structural mimicry between tRNA and domain A. Blue is core helices of domain A and red is P-site tRNA. Structures are obtained from PDB:2J00[6]. Superimposition is performed as described in the methods.
